# Mathematically Gifted Adolescents Have Deficiencies in Social Valuation and Mentalization

**DOI:** 10.1371/journal.pone.0018224

**Published:** 2011-04-04

**Authors:** Kyongsik Yun, Dongil Chung, Bosun Jang, Jin Ho Kim, Jaeseung Jeong

**Affiliations:** 1 Division of Biology, Computation and Neural Systems, California Institute of Technology, Pasadena, California, United States of America; 2 Department of Bio and Brain Engineering, Korea Advanced Institute of Science and Technology, Daejeon, Republic of Korea; 3 Department of Physics, Korea Advanced Institute of Science and Technology, Daejeon, Republic of Korea; 4 Division of Electrical Engineering, Korea Advanced Institute of Science and Technology, Daejeon, Republic of Korea; University College London, United Kingdom

## Abstract

Many mathematically gifted adolescents are characterized as being indolent, underachieving and unsuccessful despite their high cognitive ability. This is often due to difficulties with social and emotional development. However, research on social and emotional interactions in gifted adolescents has been limited. The purpose of this study was to observe differences in complex social strategic behaviors between gifted and average adolescents of the same age using the repeated Ultimatum Game. Twenty-two gifted adolescents and 24 average adolescents participated in the Ultimatum Game. Two adolescents participate in the game, one as a proposer and the other as a responder. Because of its simplicity, the Ultimatum Game is an apt tool for investigating complex human emotional and cognitive decision-making in an empirical setting. We observed strategic but socially impaired offers from gifted proposers and lower acceptance rates from gifted responders, resulting in lower total earnings in the Ultimatum Game. Thus, our results indicate that mathematically gifted adolescents have deficiencies in social valuation and mentalization.

## Introduction

Mathematically gifted adolescents are known to perform better on various cognitive tasks, including mental rotation [1], [2], problem solving [3], [4], memory processing [5], [6], and global-local processing [7]. These gifted adolescents generally have high potential to be outstanding leaders in mathematics, science, or related fields. In fact, however, many gifted adolescents are characterized as indolent, underachieving, and unsuccessful [8]. Gifted persons' above-average abilities often make it difficult for them to share their interests and to interact with others reciprocally, causing problems in social and emotional development. Thus, gifted adolescents are often judged to be emotionally maladapted to social groups [9], [10]. There are also controversial results indicating that this view of maladjustment is false [11], [12].

Although developing social valuation and mentalizing abilities in gifted adolescents is crucial for themselves and for society as a whole, few relevant studies have been done, and the results regarding the social and mentalizing skills of gifted adolescents are controversial. In this study, we aimed to quantify social valuation and mentalizing abilities in gifted adolescents using the Ultimatum Game, a simple game that can explain complex social strategic decision-making in a laboratory setting [13]. Two players participate in the game to divide a sum of money, one as a proposer and the other as a responder. The proposer decides how to divide the sum, and the responder can either accept or reject the offer. If the responder accepts, the sum is divided according to the offer. If the responder rejects, neither player obtains anything. The rational and optimal behavior, suggested by game theory, is that the proposer should offer the smallest amount possible and the responder should accept any amount offered. However, this is hardly the case in human subjects in empirical settings. On average the proposer offers 40% of the sum to the responder and 16% of the offers is rejected [14]. Because of the simplicity of the game, the Ultimatum Game is an apt tool for the investigation of complex human emotional and cognitive decision-making processes in an empirical setting.

A previous functional MRI study using the Ultimatum Game observed brain activity associated with emotion (insula) and cognition (dorsolateral prefrontal cortex) [15]. The findings imply that the relative dominance between the emotional and cognitive regions, which are responsible for fairness monitoring and economic profit-maximizing behavior, respectively, determines how humans make decisions during social interactions in the Ultimatum Game. Several studies have also found supportive evidence for emotional and cognitive processes in the Ultimatum Game [16], [17]. Another study highlighted a different aspect of the Ultimatum Game, namely the involvement of theory of mind in real social interactions [18]. This study found that the anterior paracingulate cortex and the posterior superior temporal sulcus, two of the three classic theory-of-mind areas, were activated in Ultimatum Game participants. The authors concluded that inferring the intentions of others activated the theory-of-mind neural network. Moreover, modulation of the prefrontal function using transcranial magnetic stimulation and a study of prefrontal lesion patients have been carried out in order to investigate the causal relationship between behavior and prefrontal brain activity [19], [20]. These neuroimaging studies support the idea that the Ultimatum Game is an appropriate tool for the investigation of social valuation and mentalizing abilities in gifted adolescents.

Neuroimaging studies of gifted adolescents to date have endeavored mainly to find neural correlates of their superior intelligence. Facilitated activation in the posterior parietal cortex during general intelligence tasks was found in gifted adolescents compared with controls [21]. Furthermore, when performing three-dimensional mental rotations, mathematically gifted male adolescents activate a unique brain network, including the bilateral parietal and frontal cortex, along with the anterior cingulate cortex [2]. Electroencephalography studies showed that gifted adolescents displayed higher alpha power [22], [23], more regular event-related potential waveforms [24], and less source activation [25] than average adolescents during cognitive tasks. To our knowledge, no neuroimaging studies of emotional and social abilities in gifted adolescents exist.

We hypothesized that mathematically gifted adolescents would behave deficiently in complex social decision making that requires social valuation and mind-reading abilities, described by more strategic offers, less acceptance rates, and thus less total earnings. A previous survey-based study supports our hypothesis that gifted adolescents experience difficulties in social coping strategies [26]. We analyzed the proposer and responder behaviors of gifted and average adolescents and correlated their behavior with IQ and creativity test scores. This investigation should provide insight into the social valuation and mind-reading behaviors of mathematically gifted adolescents.

## Materials and Methods

### Ethics Statement

Fully informed written consent was obtained from all subjects and their parents, and the Korea Advanced Institute of Technology Ethical Committee approved this study.

### Participants

The gifted adolescents were 22 healthy middle school volunteers (age: 14.05
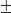
0.49 years, 16 males and 6 females) with no neurological or psychiatric diseases. They were selected through both a selective written examination and recommendations from their school principal and classroom teacher. All had been educated in a private institute with a specialized curriculum for gifted adolescents for more than two years. Twenty-four average adolescents of mean age 13.96
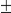
0.20 years (14 males and 10 females) from the local middle school in Daejeon, Korea, also participated. They were healthy volunteers with no history of psychiatric or neurological diseases (Table 1).

**Table 1:** Participants' demographic characteristics.

	average adolescents (N = 24)		gifted adolescents (n = 22)		Significance level
Variables	mean	SD	mean	SD	
Age (years)	13.96	0.20	14.05	0.49	t(27.20) = −0.770p = 0.448
Sex (male/female)	14/10		16/6		χ^2^ = 1.05p = 0.364
IQ	110.7	13.40	142.6	5.95	t(32.32) = −10.57* p<0.001
AKhatena-Torrance Creative Perception Inventory	56.70	18.15	66.09	23.06	t(44) = −1.560p = 0.126
What Kind of Person Are You?	48.44	24.79	66.68	27.14	t(44) = −2.408* p = 0.020
Something About Myself	64.96	29.92	65.50	30.93	t(44) = −0.061p = 0.952

A The Khatena-Torrance Creative Perception Inventory (KTCPI) score was estimated by averaging the What Kind of Person Are You (WKOPAY) and Something About Myself (SAM) scores.

*p<0.05.

The gifted adolescents showed a mean full-scale IQ of 142.6 (SD = 5.95) as measured by the Wechsler Intelligence Scale for Children-Third Edition (WISC-III) [27]. The average adolescents had a mean IQ of 110.7 (SD = 13.40) as measured by WISC-III. A Khatena-Torrance Creative Perception Inventory (KTCPI) test was also administered to both groups of adolescents [28]. The mean KTCPI score of the gifted adolescents was 66.09 (SD = 23.06) and was estimated by averaging the ‘What Kind of Person Are You’ (WKOPAY) (66.68
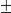
27.14) and ‘Something About Myself’ (SAM) (65.50
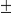
30.93) scores. In the average adolescents, the WKOPAY (48.44
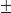
24.79) and SAM (64.96
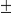
29.92) scores yielded a total KTCPI score of 56.70 (SD = 18.15). We found that the gifted adolescents had significantly higher WKOPAY and IQ scores than the average adolescents (WKOPAY: t(44) = −2.408; p = 0.020; IQ: t(32.32) = −10.57; p<0.001). Specifically, a subcategory of WKOPAY, the Disciplined Imagination (DI) score, was significantly higher in the gifted adolescents than in the average adolescents (t(44) = −2.398; p = 0.021; see Table S1). There were no significant differences in the SAM or KTCPI scores, mean age or gender (p>0.05).

### Procedures

The gifted and average groups were transported from their schools to our laboratory at KAIST. The students were distributed into two groups of proposers and responders. We confirmed that the proposers and responders did not know each other. The gifted group and the average group were tested on separate dates. In each session, two adolescents along with an instructor went into a separate room and performed the Ultimatum Game.

Two adolescents played the Ultimatum Game, one as a proposer and one as a responder, for ten trials. Each player's role was randomly assigned and fixed throughout the trials. We used face-to-face interaction to maximally shape other-regarding behaviors and a repeated game to emphasize strategic behaviors. At the beginning of the session, the subjects were given an oral explanation of the rules of the game by the instructor. Demonstration rounds were played until both of the adolescents fully understood the game. The instructor provided the proposer with ten $1 bills to begin each trial. In each trial, the proposer offered a certain portion of the $10 to the responder, who accepted or rejected the offer. During the offer, the proposer was required to explicitly count the bills one by one to prevent confusion regarding the offer amount. Then, the responder would nod up and down or shake his/her head left and right to indicate his/her decision to accept or reject the offer, respectively. If the responder accepted the offer, each player received the amount the proposer offered. If the responder rejected the offer, both players received nothing, and the money was withdrawn. The subjects were told that they could keep the money they had acquired after ten trials. The instructor recorded the offers and the responses throughout the session.

### Data Analysis

A Pearson chi-square test was used to determine whether the distributions of demographic and Ultimatum Game behavioral variables differed by group. The behavioral variables included the proposers' offer distribution, proposers' type distribution, mean offer, total earnings and the responders' acceptance rate. The offers were defined as unfair (<$5), fair ( =  $5), or hyperfair (>$5). The proposer types were either strategic or non-strategic depending on the opponent responder's decision to accept or reject the offer in the previous trial. Strategic proposers were those who raised or sustained their offer amount after their previous offer was rejected and those who lowered or sustained their offer amount after their previous offer was accepted. Non-strategic proposers were those who reduced their offer after a rejection or raised their offer after an acceptance. The alpha level was set at 0.05 for the statistical tests. Correlation analyses of the demographic and behavioral data were performed via Pearson correlations. The statistical package SPSS for Windows (version 15.0; SPSS, Inc., Chicago, IL) was used for statistical analysis.

## Results

**Figure 1:**
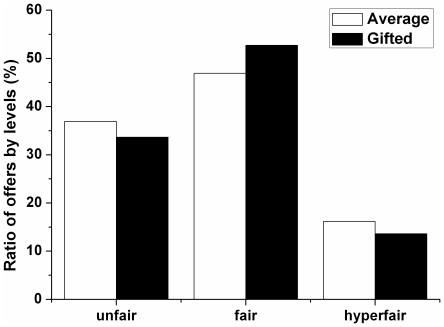
Distribution of offers made by gifted and average adolescents by level: unfair (<$5), fair ( = $5), and hyperfair (>$5).

No significant difference between the average and gifted adolescents was revealed in the distribution of offers by level (unfair, fair, and hyperfair offers) (Figure 1). In both groups, approximately 50% of the offers were fair, 35% of offers were unfair, and 15% of the offers were hyperfair. Fair offers were significantly more frequent than hyperfair offers within both groups (Average: χ^2^ = 24.798; p<0.0001; Gifted: χ^2^ = 14.02; p = 0.0002). In the average adolescents, fair offers were significantly more frequent than unfair offers (χ^2^ = 3.963 p = 0.047), but no corresponding difference was found for gifted adolescents (χ^2^ = 2.173; p = 0.140). Notably, very unfair offers ($2 or $1) were rare in both groups ($1 and $2 were each offered once in each group).

**Figure 2:**
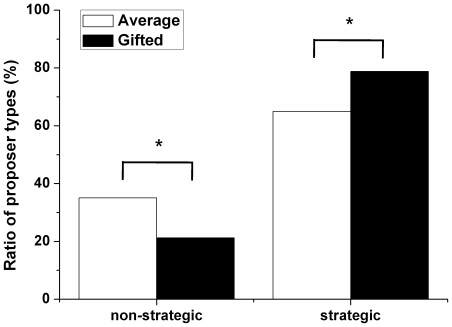
Proposer type distribution of gifted and average adolescents. A proposer offers either strategically or non-strategically depending on the opponent responder's decision to accept or reject in the previous trial. Strategic proposers were those who raised or maintained their offer amount after their previous offer was rejected or those who lowered or maintained their offer amount after their previous offer was accepted. Non-strategic proposers were those who reduced their offer after a rejection or raised their offer after an acceptance (*p<0.05, ** p<0.001).

We categorized proposers as strategic or non-strategic (Figure 2). The gifted adolescents had a significantly higher proportion of strategic proposers than did the average adolescents (χ^2^ = 4.861; p = 0.027).

The mean offer of the gifted adolescents as proposers ($4.67
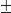
0.72) was marginally smaller than that of the average adolescents ($5.32
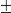
0.55), but the difference was not significant (p = 0.987).

**Figure 3:**
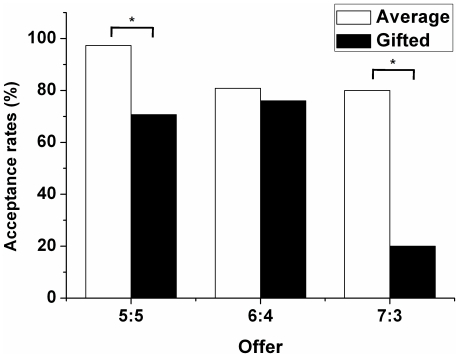
Offer acceptance rates of gifted and average adolescents (* p<0.05).

The acceptance rate of the responders in the gifted group was lower than that in the average group. Specifically, significant differences were found in the fair offer $5 (χ^2^ = 18.961; p<0.0001) and the unfair offer $3 (χ^2^ = 5.00; p = 0.025) (Figure 3).

The total earnings of the gifted adolescents ($32.95
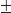
16.30) were significantly smaller than those of the average adolescents ($42.67
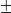
8.43) (t(30.88) = 2.57; p = 0.018).

We looked for relationships between Ultimatum Game behaviors (mean offer, distribution of offers, acceptance rate, and total earnings) and demographic variables (IQ and KTCPI). There were no correlations between IQ and behavioral variables in either the gifted or the average group (p>0.05). However, we found a significant negative correlation between the number of unfair offers and KTCPI test score in the gifted adolescents. Negative correlations were found specifically in the KTCPI subcategories of WKOPAY (r = −0.614; p = 0.034) and DI (r = −0.867; p<0.001). No significant correlations were found for the average adolescents. In addition, there were no significant differences in behavioral variables across gender.

## Discussion

In this study, we aimed to observe differences in complex social strategic behaviors between gifted and age-matched average adolescents. The results support the hypothesis that mathematically gifted adolescents behave deficiently in social valuation and theory of mind, as indicated by strategic but socially impaired offers in proposers and lower acceptance rates in responders, resulting in lower total earnings in the Ultimatum Game. These findings are consistent with previous studies of mathematically gifted adolescents that have described difficulties in social and emotional coping strategies [26]. Specifically, a study using questionnaires found that highly gifted adolescents perceive themselves as less popular, having a greater internal locus of control, and having more social and emotional problems than average adolescents [29]. In ratings of peer perceptions of athleticism, popularity and social standing, modestly gifted adolescents exceeded the highly gifted, indicating that giftedness may entail risks of developing problems in peer relations [30].

No significant difference between the average and gifted adolescents was found in the distribution of offers by level (unfair, fair, and hyperfair). Approximately half of all offers were fair in both the average and the gifted groups. These results are consistent with a previous finding that younger children made larger offers than older participants in the Ultimatum Game, suggesting that adults have made a qualitative shift to match the predictions of economic theory [31]. We speculate that both average and gifted adolescents are very sensitive to fairness. The finding that unfair $1 and $2 offers were very rare also supports this hypothesis.

The offers of the gifted adolescents were more strategic than those of the average adolescents. However, the total earnings of the gifted adolescents were lower than those of the average adolescents, indicating that strategic offer behavior does not necessarily lead to more money being earned in the game. The important aspect of the Ultimatum Game is not the mathematical strategy but rather the necessary social adaptive mentalizing strategies, including fairness, cooperation, and reputation [32]. Thus, we speculate that while gifted adolescents are mathematically more strategic, they are impaired in reading their opponent's mind.

The acceptance rate of the responders in the gifted group was lower than that of the average group for both fair and unfair offers. Rejection of unfair offers supports the hypothesis that gifted adolescents are more sensitive to unfairness and that they try to punish an opponent's unfair behavior. Rejection of a fair offer could be thought of as a highly irrational behavior. However, in the repeated Ultimatum Game with fixed players, 13% of hyperfair offers were rejected in normal adults [33]. Rejection of a fair offer in the gifted group might result from their hyper-motivation to seek a higher reward in the next trial. Consequently, their strategic behaviors made the total earnings of the gifted adolescents lower than those of the average adolescents.

We found a significant negative correlation between the ratio of unfair offers and creativity test scores in the gifted adolescents, indicating that the adolescents with better self-regulation and other-regarding behaviors offered fewer unfair and more fair proposals [28]. Negative correlations were found specifically with the KTCPI creativity test subcategories of ‘What Kind of Person are You’ and ‘Disciplined Imagination.’ These measures were designed to capture participants' perceptions of their creativity [28]. In other words, the gifted adolescents with more self-awareness of their creativity and heightened disciplined imagination were less likely to present unfair offers. These results cannot be explained by comparison with the case of the average adolescents. Previous studies have found that gifted adolescents are characterized not only by developmentally advanced electrophysiological activity of the brain, as represented by higher alpha frequency activity [22], [23], but also by distinct brain network activation, including the bilateral parietal and frontal cortices, and anterior cingulate cortex [2], [7]. Our finding of negative correlations only in the gifted and not in the average adolescents is partly explained by previous neuroimaging findings. While the creativity test scores correlated with Ultimatum Game behaviors, IQ was not correlated with any behavioral variable. The correlation results are consistent with our behavioral findings that mathematical strategies are not necessarily required for success in the game, but social adaptive mentalizing abilities are crucial.

We used face-to-face and repeated interaction with fixed players that most resemble real world ultimatum bargaining situations. The limitation is that the proposer and responder behaviors are dependent on each other. This issue could potentially be solved when the offers and responses are analyzed separately for each of the 10 trials. Thus, we computed offer fluctuations between the first and the second offer (Figure S1), ratio of offers for each trial (Figure S2) and acceptance rates for each trial (Figure S3). We found significant correlation between the first and the second offer after rejecting the first offer in the gifted group (p<0.0001). We couldn't find the significant difference between the first trial and the subsequent 9 trials in ratio of offers and acceptance rates. The results indicate that the overall offer ratio is similar between the gifted and average groups during the trials, but the distribution of offers are socially inept especially after the responder rejects the prior offer, resulted in lower acceptance rates in the gifted group. This study has provided insight into the relationship between mathematical giftedness and strategic decision making in interactive social settings. One limitation that should be considered is the relatively small number of subjects. Thus, unfair offers of $1 and $2 were very rare, and we could not perform further analysis on these offers. Furthermore, this study provides only behavioral results; thus, further research is necessary to explore the causal association between intentions and behaviors using neuroimaging techniques. We hope that this study provides insight into how gifted adolescents should be educated and how they can succeed in complex social transactions using learned social collaborative and communication skills in addition to their innate mathematical and scientific abilities.

## Supporting Information

Figure S1 Offer fluctuations between the first and the second offer. Offer fluctuation after acceptance in gifted group (Pearson correlation, p = 0.238, slope = 0.16475), rejection in gifted group (Pearson correlation, p<0.0001, slope = 0.56974), acceptance in average group (Pearson correlation, p = 0.033, slope = 0.23978), and rejection in average group (Pearson correlation, p = 0.037, slope = 0.3199).
(TIF)

Figure S2 Ratio of offers for each trial (* p<0.05).
(TIF)

Figure S3 Acceptance rates for each trial (* p<0.05).
(TIF)

Table S1 Khatena-Torrance Creative Perception Inventory statistics.
(DOCX)
