# Supplementary figures and images for: Mathematically Gifted Adolescents Have Deficiencies in Social Valuation and Mentalization
Source: PLoS One. 2011 Apr 4;6(4):e18224. doi: 10.1371/journal.pone.0018224 (PMC3070719; doi:10.1371/journal.pone.0018224)

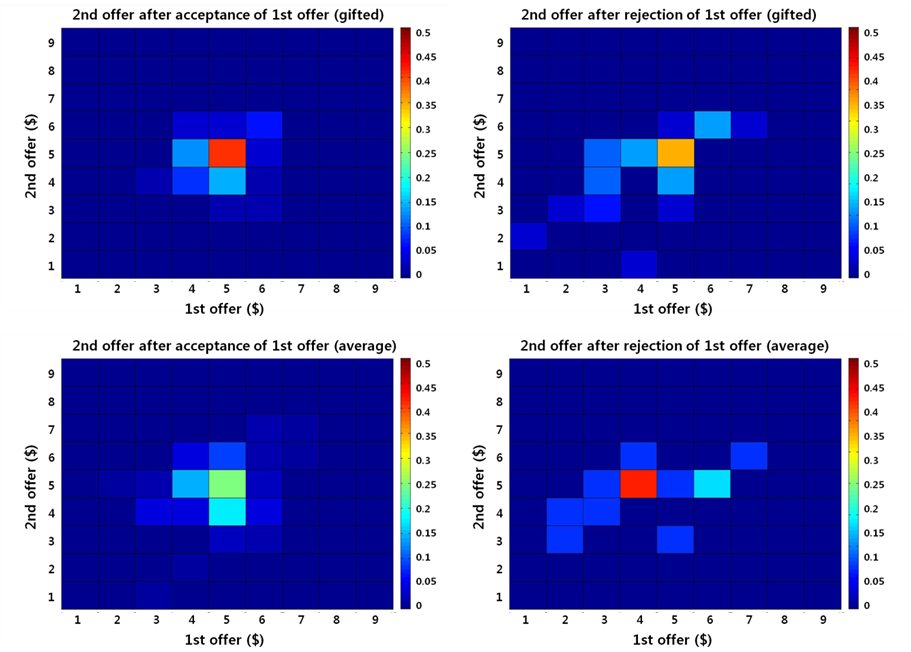

Supplement: Figure S1 — Offer fluctuations between the first and the second offer. Offer fluctuation after acceptance in gifted group (Pearson correlation, p = 0.238, slope = 0.16475), rejection in gifted group (Pearson correlation, p<0.0001, slope = 0.56974), acceptance in average group (Pearson correlation, p = 0.033, slope = 0.23978), and rejection in average group (Pearson correlation, p = 0.037, slope = 0.3199). (TIF) [file pone.0018224.s001.tif]

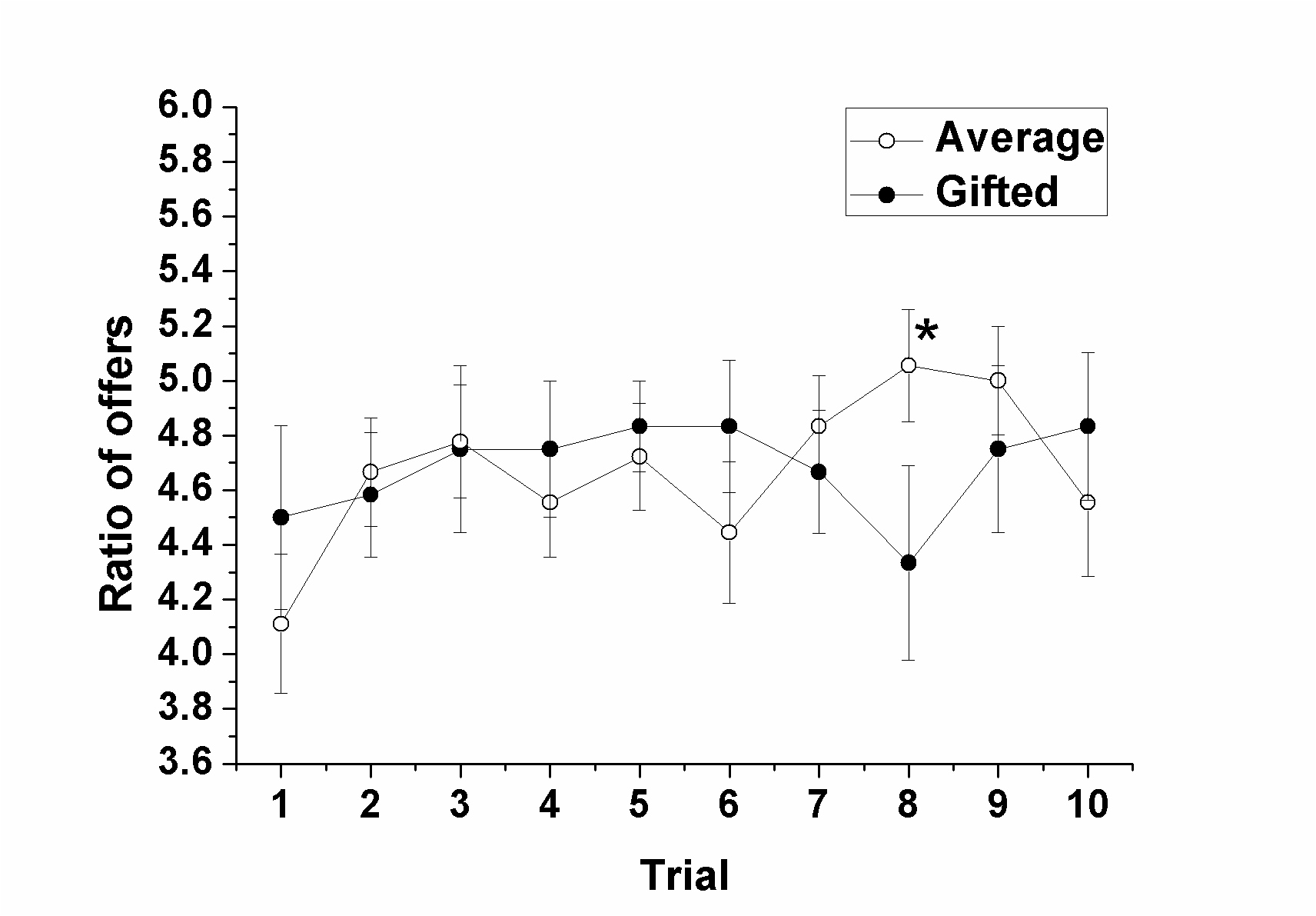

Supplement: Figure S2 — Ratio of offers for each trial (* p<0.05). (TIF) [file pone.0018224.s002.tif]

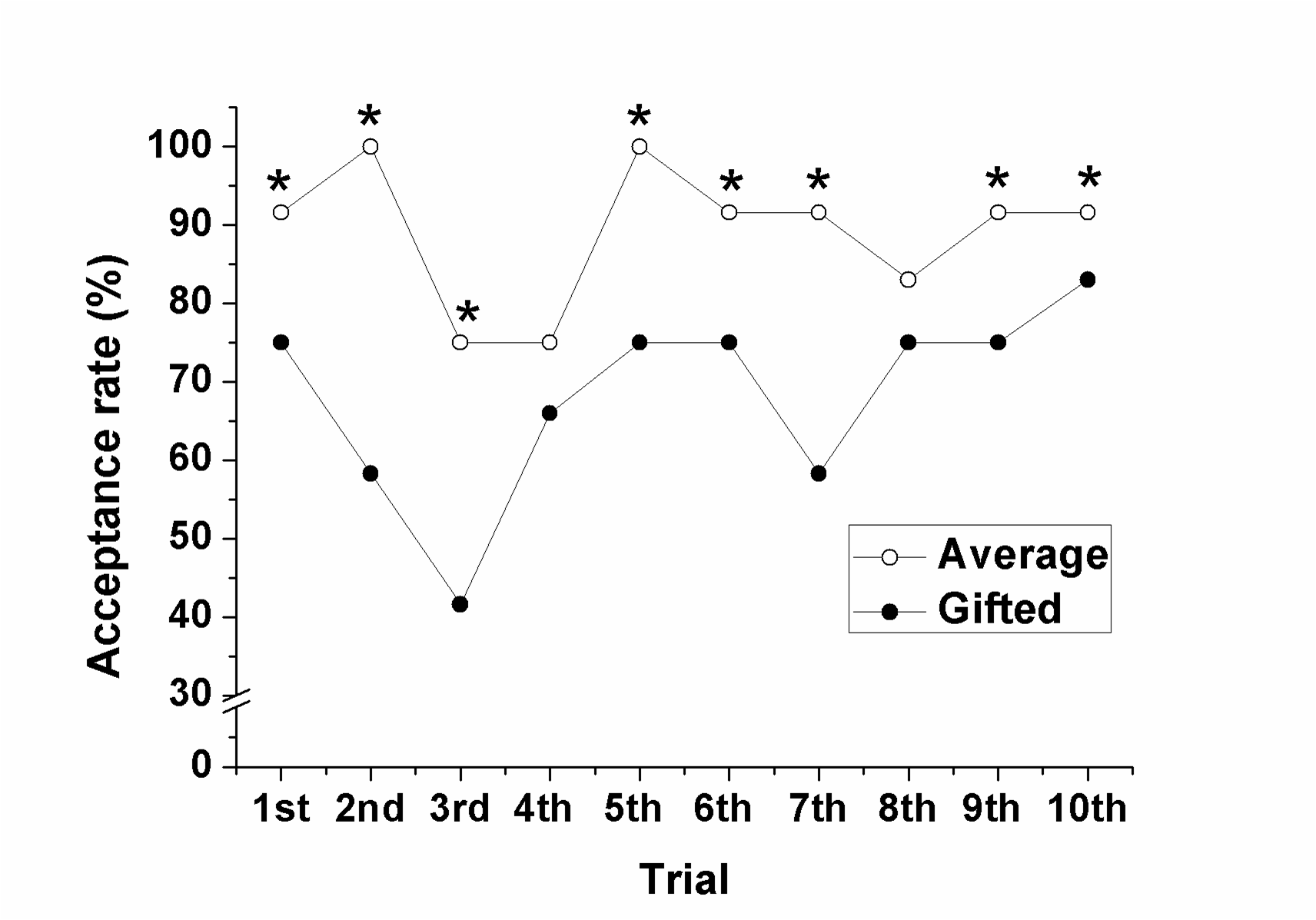

Supplement: Figure S3 — Acceptance rates for each trial (* p<0.05). (TIF) [file pone.0018224.s003.tif]
