# Supplementary material for: Mathematically Gifted Adolescents Have Deficiencies in Social Valuation and Mentalization
Source: PLoS One. 2011 Apr 4;6(4):e18224. doi: 10.1371/journal.pone.0018224 (PMC3070719; doi:10.1371/journal.pone.0018224)
Supplement: Table S1 — Khatena-Torrance Creative Perception Inventory statistics. (DOCX) [file pone.0018224.s004.docx]

**Table S1**. Khatena-Torrance Creative Perception Inventory statistics

|  | average students (N=24) | | gifted students (N=22) | |  |
| --- | --- | --- | --- | --- | --- |
| Variables | mean | SD | mean | SD | Significance level |
| **^A^**WKOPAY | 48.44 | 24.79 | 66.68 | 27.14 | t(44) = −2.408  *p = 0.020 |
| Acceptance of  authority | 57.88 | 23.24 | 48.91 | 29.00 | t(44) = 1.176  p = 0.246 |
| Self confidence | 57.72 | 32.84 | 58.41 | 26.84 | t(44) = −0.078  p = 0.938 |
| Inquisitiveness | 65.28 | 28.48 | 53.59 | 29.20 | t(44) = 1.388  p = 0.172 |
| Awareness of  others | 60.72 | 33.03 | 47.59 | 31.48 | t(44) = 1.390  p = 0.171 |
| Disciplined  imagination | 66.12 | 27.94 | 83.64 | 21.11 | t(44) = −2.398  *p = 0.021 |
| **^B^**SAM | 64.96 | 29.92 | 65.50 | 30.93 | t(44) = −0.061  p = 0.952 |
| Environmental  sensitivity | 50.60 | 32.66 | 53.23 | 37.18 | t(44) = −0.258  p = 0.798 |
| Initiative | 66.44 | 25.64 | 65.18 | 27.74 | t(44) = 0.162  p = 0.872 |
| Self strength | 70.12 | 27.28 | 68.91 | 25.89 | t(44) = 0.155  p = 0.877 |
| Intellectuality | 62.84 | 30.74 | 61.41 | 32.34 | t(44) = 0.155  p = 0.877 |
| Individuality | 54.92 | 32.20 | 66.91 | 25.13 | t(44) = −1.431  p = 0.159 |
| Artistry | 52.48 | 35.06 | 45.82 | 32.46 | t(44) = 0.673  p = 0.505 |

**^A^** What Kind of Person are You, **^B^** Something about Myself, *p < 0.05
